# A Meshfree Method for Simulating Myocardial Electrical Activity

**DOI:** 10.1155/2012/936243

**Published:** 2012-09-03

**Authors:** Heye Zhang, Huajun Ye, Wenhua Huang

**Affiliations:** ^1^Shenzhen Institutes of Advanced Technology, Chinese Academy of Science, Shenzhen 518055, China; ^2^Department of Optical Engineering, Zhejiang University, Hangzhou 310027, China; ^3^Institute of Clinical Anatomy, Southern Medical University, Guangzhou 510515, China

## Abstract

An element-free Galerkin method (EFGM) is proposed to simulate the propagation of myocardial electrical activation without explicit mesh constraints using a monodomain model. In our framework the geometry of myocardium is first defined by a meshfree particle representation that is, a sufficient number of sample nodes without explicit connectivities are placed in and inside the surface of myocardium. Fiber orientations and other material properties of myocardium are then attached to sample nodes according to their geometrical locations, and over the meshfree particle representation spatial variation of these properties is approximated using the shape function of EFGM. After the monodomain equations are converted to their Galerkin weak form and solved using EFGM, the propagation of myocardial activation can be simulated over the meshfree particle representation. The derivation of this solution technique is presented along a series of numerical experiments and a solution of monodomain model using a FitzHugh-Nagumo (FHN) membrane model in a canine ventricular model and a human-heart model which is constructed from digitized virtual Chinese dataset.

## 1. Introduction

 Myocardial contraction is driven by a sequence of propagating electrical activations throughout the myocardium [1]. Propagation of electrical activations inside the myocardium is a highly complicated process mainly due to the fibrous structure of myocardium, as shown in many experiments [2]. There have been efforts in simulating myocardial electrical activations using computational models with known physical parameters, including the source intensities and locations, material properties, and boundary conditions, because these simulations can help to understand the measurement data, suggest new experiments, and provide insights into the basic mechanism of electrical activity in the heart. A number of computational models have been developed to simulate the macroscopic electrical propagation process [3, 4], such as cellular automata and reaction-diffusion systems. A cellular automaton is a discrete model which usually consists of a regular grid of cells, each in one of a finite number of states. Every cell has the same rule for updating, based on the states in its neighborhood. Because the simplicity of states and superior computational speed resulted from rules, cellular automata have been applied in simulations of myocardial electrical activity in the heart [5, 6], but such simplistic and rule-based approaches cannot always properly capture the shape of transmembrane potentials. A reaction-diffusion system is a mathematical model that describes how the concentration of one or more substances distributed in space changes under the influence of two processes: local reactions in which the substances are converted into each other and diffusion which causes the substances to spread out in space. This concept in the reaction-diffusion system is borrowed and applied in the simulation of myocardial electrical activity by turning local reactions into cellular models, that is, ionic currents, and diffusion into transmission of transmembrane potentials, that is, anisotropic propagation through myofibers. Though the reaction-diffusion system can more appropriately reproduce electrical activity of excitable myocardium [3, 4, 7] than cellular automata, solving a reaction-diffusion system is computationally expensive with realistic modelling of cardiac tissue properties and cellular behaviors [3]. Recently, the Eikonal model [8, 9], which is a simplified wavefront model, has also been solved by FEMs in order to simulate anisotropic electrical activity across myocardium [10]. The computational models have been widely applied in understanding patients' data [11–15].

In the context of modelling electrical activity in the heart, one of challenges is to establish a numerical representation of the complex geometry of the heart. This representation must not only characterize geometric complexities but also be of sufficient resolution to capture the activation wavefront and perhaps cellular behaviours. In order to properly simulate myocardial electrical activity by solving reaction-diffusion systems accurately, a large number of numerical schemes have been developed by representing the intrinsic structure of the myocardium and the inhomogeneity/anisotropy in different ways. By discretizing the diffusion tensor over the problem domain, traditional FDMs can evolve electrical activity over orthogonal and regular grids [16], but the complex geometry of heart is always a great challenge for FDMs. Thus, a few works have proposed the use of irregular grids with FDMs to deal with complex geometry by increasing the complexity of interpolation between grids [7, 17–19]. In FEMs, the integral form of the reaction-diffusion system is discretized over a finite element representation of the geometry. Typically low-order (linear Lagrange basis) elements used in FEMs [20, 21] always lead to a high number of elements in the complex geometry and a long time integration for a certain accuracy or remesh in changing geometry, such as a beating heart. Therefore, high-order elements, such as cubic Hermite basis elements [22] and quadratic Lagrange basis elements, use more nodal parameters or nodes inside one element to get better accuracy, but the size of system matrix and the computational load are also increased largely. Furthermore, meshing or remeshing for FEMs using high-order elements still remains challenge.

EFGM is developed as a meshfree method in 1990s [23] and has been successfully applied for a wide range of mechanical applications [24, 25]. A series of publications [26, 27] have explored the numerical capabilities of EFGM, including parallelization and comparison with FEMs in mechanical applications. Meshfree method has been applied into simulation of myocardial electrical activity by authors [28, 29]. However, the numerical performance of meshfree method has not been well verified in the simulation of myocardial electrical activity. Furthermore, the previous work [28, 29] only used left ventricle segmented from MR images with spurious fiber structure. Our aim of this paper is to present EFGM as a computational tool to solve reaction-diffusion systems for the simulation of myocardial electrical activity. In this paper a new representation of myocardial geometry and fiber structure by a cloud of nodes without any explicit connectivity defined between them, that is, meshfree particle representation, is first discussed. Upon this representation, the numerical performance of EFGM in solving the monodomain model [3, 4], a reaction-diffusion system, is demonstrated through experiments. The properties processed by EFGM provide quite a few advantages, such as refinement can be accomplished by adding or removing nodes in particular areas [23–25]. Moreover, fiber orientation is interpolated with nodal parameters, not inside the element any more. Hence, all the operations inside the element of FEMs, such as coordinate transform from elemental coordinate to global coordinate and the interpolation of elemental fiber orientation, are also avoided in this approach. Furthermore, higher order approximation of a meshfree shape function can be achieved without rearranging nodal positions or adding extra degrees of freedom in nodes for example, higher consistency and continuity can be still maintained over the whole problem domain, even with a linear basis, in EFGM [23–25]. Though it has been demonstrated that EFGM can also handle material inhomogeneities and discontinuities in mechanical applications [24, 25], we cannot cover that in this paper because of limited space.

Governing equations of electrical activity over the myocardium are discussed in Section 2. A numerical scheme based on EFGM in terms of representation, shape function and the Galerkin weak form is established in Section 3. Numerical experiments are presented and compared in Section 4. And finally in Section 5, we discuss the strengths and weaknesses of the current approach and state possible future directions.

## 2. Governing Equations

The bidomain model [3, 4], a popular reaction-diffusion system, divides the myocardium into intracellular and extracellular space. Both spaces can be described by the same coordinate system and are separated by the membrane at each location:

(1) $$\begin{array}{c} ▿\cdot \left(\left(D_{i}+D_{e}\right)▿v_{e}\right)=-▿\cdot \left(D_{i}▿v_{m}\right)+I_{s1}, \end{array}$$

(2) $$\begin{array}{c} ▿\cdot \left(D_{i}▿v_{m}\right)+▿\cdot \left(D_{i}▿v_{e}\right)=A_{m}\left(C_{m}\frac{\partial v_{m}}{\partial t}+I_{\text{ion}}\right)-I_{s2}. \end{array}$$

*v*_*m*_ is the transmembrane potential, *v*_*e*_ is the extracellular potential, **D**_*i*_ is the conductivity in intracellular space, **D**_*e*_ is the conductivity in extracellular space, *A*_*m*_ is the ration of the membrane surface area to the volume, *C*_*m*_ is the membrane capacitance, *I*_ion_ is sum of ionic currents, and *I*_*s*1_ and *I*_*s*2_ are external stimulus currents. There are a lot of cellular ionic models [3, 4] that could be used in reaction-diffusion system. If the conductivity in extracellular domain is assumed to be infinitely large, or the conductivities of extracellular and intracellular domains are assumed to be equally anisotropic, for example, **D**_*i*_ = *k* · **D**_*e*_, a bidomain model can be reduced to a monodomain model, which turns (1) and (2) into a single equation:

(3) $$\begin{array}{c} ▿\cdot \left(D▿v_{m}\right)=A_{m}\left(C_{m}\frac{\partial v_{m}}{\partial t}+I_{\text{ion}}\right)-I_{s}, \end{array}$$

with natural boundary condition (**D**▿*v*_*m*_) · **n** = 0 if heart is considered as an isolated continuum. *I*_*s*_ is external stimulus current, and **D** is the conductivity. The conductivity variables, **D**_*e*_, **D**_*i*_, or **D**, at each point in space, are represented by a tensor containing coefficients along and across fiber orientation in that point. Let **D**_local_ be a diffusion tensor of a point in local fiber coordinate; then in 3D

(4) $$\begin{array}{c} D_{\text{local}}=\left(\begin{array}{ccc} \sigma_{f} & 0 & 0\\ 0 & \sigma_{\text{cf}} & 0\\ 0 & 0 & \sigma_{\text{cf}} \end{array}\right), \end{array}$$

where *σ*_*f*_ along the fiber and *σ*_*cf*_ across fiber. In some three-dimensional simulation works, directions of sheet of fiber and cross-sheet of fiber are treated differently [22, 30].

Hence the **D** of one point with *α* and *β* defining a rotation around the *z*- and *y*-axis of the global coordinate system according to the fiber orientation can be defined:

(5) $$\begin{array}{c} D=TD_{\text{local}}T^{-1},\;\;\;\;\;\;\;T=R_{xz}R_{xy}, \end{array}$$

(6) $$\begin{array}{c} R_{xy}=\left(\begin{array}{ccc} \cos\alpha & \sin\alpha & 0\\ -\sin\alpha & \cos\alpha & 0\\ 0 & 0 & 1 \end{array}\right)\;\;R_{xz}=\left(\begin{array}{ccc} \cos\beta & 0 & \sin\beta \\ 0 & 1 & 0\\ -\sin\beta & 0 & \cos\beta \end{array}\right). \end{array}$$

## 3. Element-Free Galerkin Method

The reaction-diffusion system is a dynamic system controlled by the diffusion term and reaction term. However, there are too many cellular models, that is, reaction terms, which are beyond the scope of this paper. Therefore, we choose the monodomain model with polynomial cellular model to verify the numerical performance of EFGM in this paper: meshfree particle representation of geometry and fiber structure by unstructured nodes is established first, and then meshfree shape function is constructed from those unstructured nodes; after obtaining Galerkin weak form of the monodomain model using meshfree shape function over meshfree particle representation, a regular background mesh, served as an integration scheme, is applied to solve Galerkin weak form of the monodomain model numerically.

### 3.1. Meshfree Particle Representation

**Figure 1:**
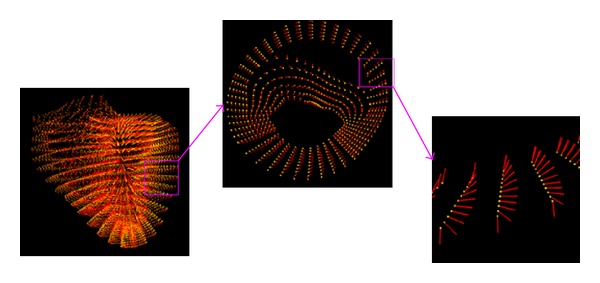
Meshfree representation of Auckland heart model.

 In FEMs the problem domain is always discretized by finite elements, such as triangular meshes in 2D and tetrahedral meshes in 3D. These elements are constructed through certain constraints, such as connectivity and size. Then field variables, such as potential or fiber direction, are interpolated by elemental shape function. However in EFGM the problem domain is represented by a cloud of unstructured nodes without any predefined connectivity, named by meshfree particle representation, and field variables are approximated by meshfree shape function. In Figure 1, a meshfree representation of Auckland heart model and its fiber orientations is shown from whole view, one slice to one section of muscle wall. In meshfree particle representation all nodal positions can be arbitrary, so irregular boundaries or interfaces of inhomogeneity can be simply represented by nodes and nodal positions can follow the changing of boundaries or interfaces easily [23–25]. Several works also developed different adaptive meshfree representations using level set method [31], triangular meshing in 2D [25], or tetrahedral meshing in 3D [25]. Moreover, refinement of EFGM could be accomplished by adding or removing nodes into existing representation according to the requirement of accuracy in interested area, such as more nodes should be added into interested area if the error is particularly large or higher accuracy is required in this local area [24, 25].

### 3.2. Meshfree Shape Function

 After meshfree particle representation is established, approximation of field variables can be computed using meshfree shape function and finite nodal values. Construction of shape function is the kernel of EFGM, which includes three steps: (1) determine the size of influence domain of each Gaussian point and search nodes (In this paper, the node only refers to the node of the meshfree representation, Gaussian point always refers to the quadrature point of Gaussian quadrature scheme.) which fall inside the influence domain of Gaussian point from meshfree particle representation, for example, **x**_*I*_  (In this paper, **x**_*i*_ refers to index of coordinates, and **x**_*I*_ refers to the index of nodes) and *I* = 1,…, *n*; (2) choose proper weighting parameters and calculate weight function; (3) compute entries of meshfree shape function and its derivatives in the position of each Gaussian point using moving least square (MLS) approximation.

#### 3.2.1. Influence Domain

**Figure 2:**
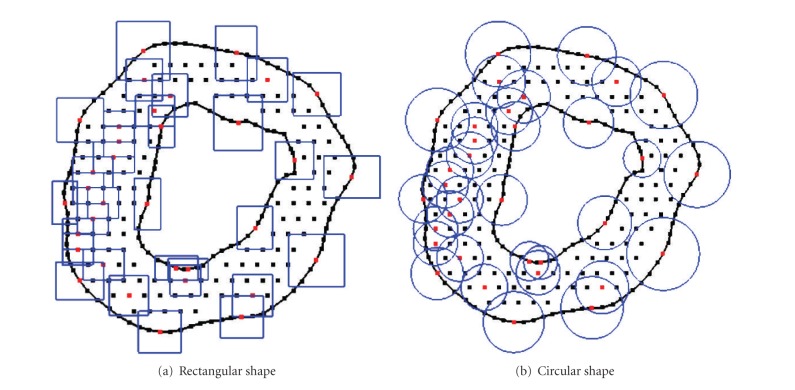
Examples of influence domains in 2D. (a) rectangular shape and (b)circular shape.

 The influence domain is used to determine an influence area/supporting area of one point, usually Gaussian point, inside the meshfree particle representation. The shape of influence domain can be any arbitrary closed shape in space, while circle or rectangle in two dimensions and sphere or cube in three dimensions are commonly used [24, 25]. Examples of circle and rectangle influence domains are shown in Figure 2. The size of influence domain should reflect the density of nodes (i.e., the size of influence domain in coarse area should be large and the size of influence domain in dense area should be small), and besides, influence domain of one point has to be overlapped with influence domains of neighbouring points to guarantee a smooth approximation of field variables and their derivatives (*C* ^1^ continuity). The size of influence domain of node **x**_*I*_, *d*_*mI*_, is calculated as

(7) $$\begin{array}{c} d_{mI}=d_{\max}c_{I}, \end{array}$$

where *d*_max⁡_ is a scaling parameter which might vary between different applications and could be determined by numerical experiments [23, 25]. The distance *c*_*I*_ is determined by searching enough neighbouring nodes for the matrix **A** in (22), which is discussed in the following subsection, to be invertible, which is also a good strategy to reflect the density of nodes. But influence domain of a point near to any discontinuity should be cut by the discontinuity, including boundary, if this influence domain crosses the discontinuity during the construction of meshfree shape function [24, 25, 32], because the nodes in one side of discontinuity could not affect the nodes or area in the other side of discontinuity. Though cardiac tissue is discontinuous and fiber orientations will not change smoothly at a certain scale any more [3], the heart still could be modelled as a continuum for the propagation of electrical activity, which will not damage our purpose to demonstrate the numerical performance of EFGM.

#### 3.2.2. Weight Function

 The weight function, a function of distance ||**x** − **x**_*I*_||, which obtains a compact support from the influence domain, needs to be positive to guarantee all meshfree shape functions unique, smooth and continuous throughout the entire problem domain to fulfill the compatibility requirement so that the nodes further from **x** will have smaller weights [23–25]. Cubic weight function and quartic weight function are popularly used and they can be replaced by each other in EFGM without rearrangement of nodal positions. Cubic weight function is

(8) w(||x−xI||dmI)  ≡w(rI)={23−4rI2+4rI3  for  rI≤12,43−4rI+4rI2−43rI3for  12<rI≤1,0for  rI>1,

where *r*_*I*_ = ||**x** − **x**_*I*_||/*d*_*mI*_. And the spatial derivative of cubic weight function in location **x** is:

(9) $$\begin{array}{c} \frac{dw\left(r_{I}\right)}{dx_{i}}=\frac{dw\left(r_{I}\right)}{dr_{I}}\frac{dr_{I}}{dx_{i}}\{\begin{array}{cc} \left(-8r_{I}+12r_{I}^{2}\right)\frac{dr_{I}}{dx_{i}} & \text{for}\;\;r_{I}\leq \frac{1}{2}\text{,}\\ \left(-4+8r_{I}-4r_{I}^{2}\right)\frac{dr_{I}}{dx_{i}} & \text{for}\;\;\frac{1}{2}<r_{I}\leq 1,\\ 0 & \text{for}\;\;r_{I}>1. \end{array} \end{array}$$

Quartic weight function is

(10) $$\begin{array}{c} w\left(\frac{||x-x_{I}||}{d_{mI}}\right)\equiv w\left(r_{I}\right)=\{\begin{array}{cc} 1-6r_{I}^{2}+8r_{I}^{3}-3r_{I}^{4} & \text{for}\;\;r_{I}\leq 1,\\ 0 & \text{for}\;\;r_{I}>1, \end{array} \end{array}$$

where *r*_*I*_ = ||**x** − **x**_*I*_||/*d*_*mI*_. And the spatial derivative of quartic weight function in location **x** is

(11) dw(rI)dxi =dw(rI)drIdrIdxi{(−12rI+24rI2−12rI3)drIdxifor  rI≤1,0for  rI>1.

#### 3.2.3. MLS Approximation

 Let *u* ^*h*^(**x**) be the approximation of state variable *u*_**x**_ at point **x**. In the MLS approximation:

(12) $$\begin{array}{c} u^{h}\left(x\right)=\sum_{i=1}^{m}p_{i}\left(x\right)e_{i}\left(x\right)\equiv P^{T}e, \end{array}$$

where *p*_*i*_(*x*) are the polynomial basis functions, *m* the number of terms in the basis functions, and *e*_*i*_(*x*) the unknown coefficients which will be determined later. The basis functions usually consist of monomials of the lowest orders to ensure minimum completeness, and common ones are linear basis:

(13) pT={1,x} in  1D,  pT={1,x,y} in  2D,pT={1,x,y,z} in  3D,

and the quadratic basis:

(14) $$\begin{array}{c} p^{T}=\left\{1,x,x^{2}\right\}\;\text{in}\;\;1\text{D},\\ p^{T}=\left\{1,x,y,x^{2},xy,y^{2}\right\}\;\text{in}\;\;2\text{D},\\ p^{T}=\left\{1,x,y,z,x^{2},y^{2},z^{2},xy,xz,yz\right\}\;\text{in}\;\;3\text{D}. \end{array}$$

In EFGM, **P** ^*T*^ in (12) can be replaced by any other polynomial basis **P** ^*T*^ without the rearrangement of nodal positions [24, 25]. The consistency of the MLS approximation depends on the complete order of polynomial basis **P** ^*T*^. If the complete order of polynomial basis, **P** ^*T*^, is *m*, the meshfree shape function will possess *C* ^*m*^ consistency [24, 25].

Given a set of *n* nodal values *u*(*x*_1_), *u*(*x*_2_),…, *u*(*x*_*n*_) of the field variable *u* at a set of nodes {**x**_*I*_} = *x*_1_, *x*_2_,…, *x*_*n*_. The coefficients *a*_*i*_(*x*) are obtained by minimizing the difference between the local approximation *u* ^*h*^(**x**) and the actual nodal parameter *u*(**x**_*I*_) in location **x**:

(15) J=∑I=1nw(rI)[uh(x)−u(xI)]2=∑I=1nw(rI)[∑i=1mpi(xI)ei(x)−u(xI)]2,

where *w*(*r*_*I*_) is the weighting function with compact support within the influence domain, which is defined in Section 3.2.2 Equation (15) can be rewritten into matrix form:

(16) $$\begin{array}{c} J={\left(Pe-u\right)}^{T}W\left(Pe-u\right), \end{array}$$

where

(17) u=[u1,u2,…,un]T,P=[p1(x1)p2(x1)⋯pm(x1)p1(x2)p2(x2)⋯pm(x2)⋮⋮⋱⋮p1(xn)p2(xn)⋯pm(xn)],W=[w(r1)0⋯00w(r2)⋯0⋮⋮⋱⋮00⋯w(rn)].

At point **x**, coefficients **E**(**x**) are chosen by minimizing the weighted residual, which are realized through ∂*J*/∂*e* = 0:

(18) $$\begin{array}{c} \frac{\partial J}{\partial e}=AE-Bu=0 \end{array}$$

therefore,

(19) $$\begin{array}{c} e=A^{-1}Bu, \end{array}$$

where

(20) $$\begin{array}{c} A=P^{T}\left(x_{I}\right)W\left(r_{I}\right)P\left(x_{I}\right),\;\;B=P^{T}\left(x_{I}\right)W\left(r_{I}\right). \end{array}$$

Substituting (19) into (12), the approximation *u* ^*h*^(**x**) becomes

(21) $$\begin{array}{c} u^{h}\left(x\right)=\sum_{I=1}^{n}\phi_{I}\left(x\right)u_{I}=\phi \left(x\right)u, \end{array}$$

where the meshfree shape function *ϕ*(**x**) is defined by

(22) $$\begin{array}{c} \phi \left(x\right)=P{\left(x\right)}^{T}A^{-1}B, \end{array}$$

with *m* the order of the polynomial in **P**(**x**). Note that *m*, the number of terms of the polynomial basis, is usually set to be much smaller than *n*, the number of nodes used for constructing the meshfree shape function. The spatial derivative of meshfree shape function in **x** is obtained by:

(23) ϕ(x),xi=(PT(x)A−1B),xi=P,xiT(x)ATB+PT(x)A,xiTB    +PT(x)ATB,xi,

where

(24) $$\begin{array}{c} B_{,x_{i}}=\frac{dW\left(r_{I}\right)}{dx_{i}}P\left(x_{I}\right), \end{array}$$

and **A**_,*x*_*i*__^−1^ is computed by

(25) $$\begin{array}{c} A_{,x_{i}}^{-1}=-A^{-1}A_{,x_{i}}A^{-1},\;\;A_{,x}=P^{T}\left(x_{I}\right)\frac{dW\left(r_{I}\right)}{dx_{i}}P\left(x_{I}\right), \end{array}$$

where *d* **W**(*r*_*I*_)/*dx*_*i*_ is defined in Section 3.2.2 Then the approximation of first derivative of field variable *u* can be obtained in **x**:

(26) $$\begin{array}{c} u^{h}{\left(x\right)}_{,x_{i}}=\sum_{i=1}^{N}\phi {\left(x\right)}_{,x_{i}}u_{I}, \end{array}$$

and is continuous in the whole problem domain.

### 3.3. Construction of Galerkin Weak Form

 In Galerkin weak form differential equations are transformed into integral form by using the weighted residual strategies so that they are satisfied over a domain in an integral sense rather than every point. Consider the integral form of (3), which also can be easily applied to bidomain (1), we have

(27) $$\begin{array}{c} \int_{\Omega }\left[\nabla \cdot \left(D\nabla v_{m}\right)-A_{m}\left(C_{m}\frac{\partial v_{m}}{\partial t}+I_{\text{ion}}\right)\right]\nu d\Omega =0, \end{array}$$

where *ν* is the trial function. The exact solution of (3) should always satisfy integral in (27). Substituting *f* = −*A*_*m*_ *I*_ion_ and evaluating integral in (27) using Green's formulae

(28) ∫ΩAmCm∂vm∂tνdΩ+∫Ω∇vm·D∇νdΩ−D∮Sν∂vm∂ndS  =∫ΩfνdΩ,

where *S* is the boundary of *Ω* and **n** is a vector normal to boundary. Equation (28) can automatically fulfill zero natural boundary condition, ∂*v*_*m*_/∂**n** = 0, by eliminating **D**∮_*S*_ *ν*(∂*v*_*m*_/∂**n**)*dS* at boundary *S*, but an accurate numerical integral scheme should be applied to the rest parts of (28) so that zero natural boundary condition can be enforced correctly in numerical sense. In Galerkin weak form procedure, trial function could be replaced by the shape function, Φ^*T*^, of EFGM here:

(29) $$\begin{array}{c} \int_{\Omega }A_{m}C_{m}\frac{\partial v_{m}}{\partial t}\Phi^{T}d\Omega +\int_{\Omega }\nabla v_{m}D\nabla \Phi^{T}d\Omega =\int_{\Omega }f\Phi^{T}d\Omega . \end{array}$$

To solve (29) we need to discrete them. Let **v**_*I*_ be the vector of nodal values of transmembrane potentials *v*_*m*_, and let **f**_*I*_ be the vector of nodal values of *f* = −*A*_*m*_ *I*_ion_ at node set **x**_*I*_. Then *v*_*m*_ ≈ Φ**v**_*I*_ and *f* ≈ Φ**f**_*I*_ and a continuous form of (29) can be discretized:

(30) AmCm∂vI∂t∫ΩΦΦTdΩ+DvI∫Ω∇Φ∇ΦTdΩ  =fI∫ΩΦΦTdΩ.

Rewrite equations previously mentioned with matrices:

(31) $$\begin{array}{c} A_{m}C_{m}\frac{\partial v_{I}}{\partial t}+M^{-1}Kv_{I}=f_{I},\\ M_{i,j}=\int_{\Omega }\phi_{i}^{T}\phi_{j}d\Omega ,\;\;K_{i,j}=\int_{\Omega }B_{i}^{T}DB_{j}d\Omega \\ B_{i}=\left[\begin{array}{c} \phi_{i,x}\\ \phi_{i,y}\\ \phi_{i,z} \end{array}\right], \end{array}$$

with **D** the diffusion tensor transformed from fiber coordinate (5), *ϕ*_*i*,*x*_, *ϕ*_*i*,*y*_, and *ϕ*_*i*,*z*_ the derivatives of the shape function with respect to *x*, *y*, and *z*, *ϕ*_*i*_ the matrix of shape functions, and **B**_*i*_ the differential matrix at the *i*th node.

### 3.4. Integration Schemes

The shape function of EFGM does not fulfill the property of strict interpolation, that is, *ϕ*_*i*_(*x*_*j*_) ≠ *δ*_*ij*_, which implies that essential boundary condition cannot be imposed directly, so penalty method and Lagrange multiplier are proposed to enforce essential boundary condition in EFGM [32, 33]. However, zero natural boundary condition can be enforced in Galerkin weak form (Equation (28)) by placing sufficient nodes along the boundaries and then applying a correct integration scheme.

**Figure 3:**
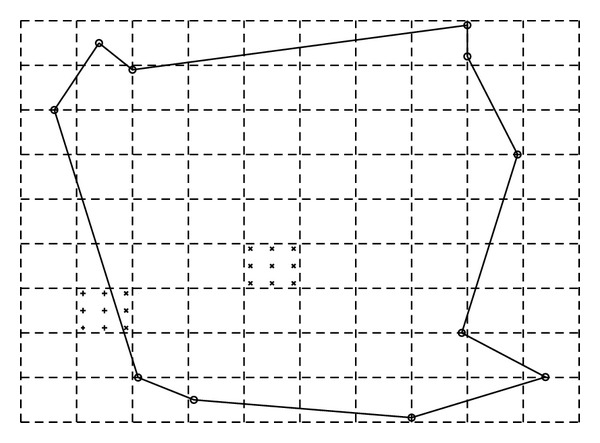
Dashed square cells consist of a background mesh, which covers the whole problem domain—the area confined by solid lines. In each cell, Gauss quadrature will be applied. Here only two cells are marked to illustrate this process. Those Gauss points, indicated by × marker, which are inside problem domain will be counted during integration, but the other Gauss points, indicated by + marker, which are out of problem domain, will not be counted.

In EFGM, a regular background mesh, which consists of nonoverlapping regular cells and covers the whole problem domain, is a very popular choice to perform the integration of computing **M** and **K** matrix in (29) because of its simplicity. The regular cells of background mesh are commonly squares in two dimension, and cubes in three dimensions. The proper density of background mesh needs to be designed to approximate solutions of desired accuracy. In each cell, Gauss quadrature scheme is used. The number of quadrature points, integration points, seems to depend on the number of nodes in the cell. An empirical guideline of quadrature points suggests [25]

(32) $$\begin{array}{c} n_{q}=\sqrt{n_{n}}+2\;\text{in}\;\;2\text{D},\;\;n_{q}=\sqrt[{3}]{n_{n}}+1\;\text{in}\;\;3\text{D}, \end{array}$$

where *n*_*n*_ is the number of nodes in the cell and *n*_*q*_ is the number of quadrature points in one cell. Our experience with Gauss quadrature in EFGM suggests that a lower order quadrature (smaller *n*_*q*_) with finer background mesh may be preferable to a higher order quadrature (larger *n*_*q*_) with coarser background mesh. The background cells are usually independent of the arrangement of sample nodes and large enough to hold the whole problem domain, but in regular domain with regular nodes, it can be coincided with problem domain and depend on nodal positions. In the background mesh, there may exist the cell that does not entirely belong to the problem domain; that is, only a portion of this cell would contribute to (29). This contribution could be realized by counting the quadrature weights of those quadrature points in this cell, which are inside problem domain, and ignoring other quadrature points of this cell, which are outside problem domain (Figure 3). Therefore, a scheme that automatically detects the quadrature points of each cell which lie inside of the problem domain is employed. Hence the integral of (29) over irregular problem domain is solved numerically in those quadrature points inside problem domain. In [26], an irregular background mesh is proposed to achieve higher accuracy, but the improvement is not obvious and it may increase the time of assembling system matrices. Finally we can give out the flow of EFGM:

1. set up sample nodes,
2. set up background mesh and quadrature points in all cells,
3. loop over all the quadrature points,
  - if this quadrature point is outside the problem domain, go to 3*e, *
  - determine nodes whose influence domains cover this quadrature point by searching enough neighboring nodes,
  - calculate quadrature weight, weight function and shape function in this quadrature point,
  - assemble **M** and **K** matrices in (29),
  - end if,
4. End loop.

## 4. Experiment

 Numerical experiments are implemented by Matlab, and the simulation of electrical propagation in Auckland heart model is implemented by C++ and Matlab external C++ math library in a Dell precision T3400 workstation with a quad cores 2.4 GHz CPU and 4G DDR2 memory. Let Π_*i*_^exact^ be the analytical solution and let Π_*i*_^numerical^ be the numerical solution in node *i*, respectively. To a set of nodes, from 1 to *N*, root mean square (RMS) error is.

(33) $$\begin{array}{c} \text{RMS}=\sqrt{\frac{1}{N}\sum_{i=1}^{N}{\left(\Pi_{i}^{\text{exact}}-\Pi_{i}^{\text{numerical}}\right)}^{2}.} \end{array}$$

**Figure 4:**
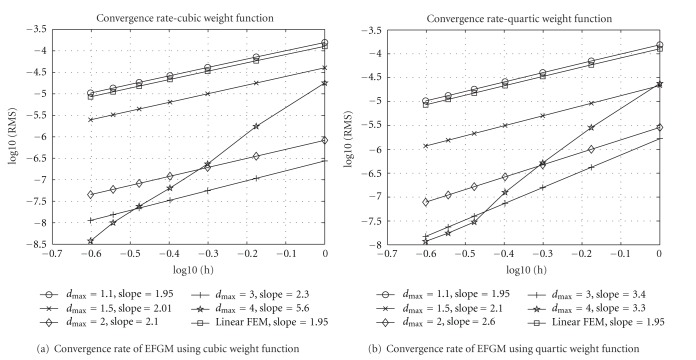
All the experiments, including FEM, use linear polynomial basis, and their convergence rates are indicated by slope. All the nodes are regularly placed and so *h* is the spacing between nodes. 4 × 4 Gaussian quadrature scheme in each cell of background mesh is used for numerical integration.

**Figure 5:**
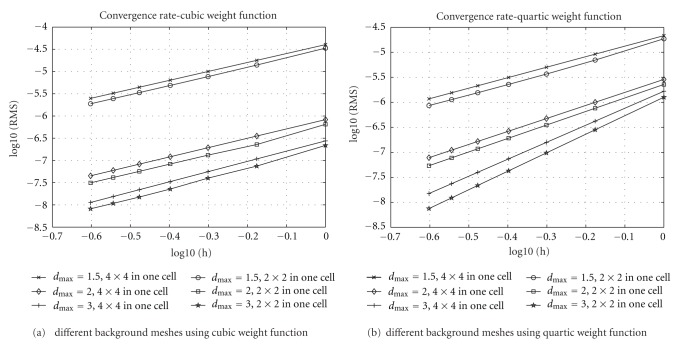
Convergence behaviours of EFGM using different background mesh. *h* is the spacing between nodes. RMS is the error measure. It shows that 2 × 2 Gaussian quadrature scheme in each cell of fine background mesh works a little better than 4 × 4 Gaussian quadrature scheme in each cell of coarse background mesh.

The behaviour of reaction-diffusion equation is controlled by the diffusion term and reaction term simultaneously. The reaction term could have huge varieties in electrical propagation applications [3, 4], and it is impossible to evaluate EFGM's performance over all the forms of reaction term in this paper; however it would be valuable to compare EFGM to FEM in approximating diffusion process. So a two-dimensional heat conduction problem without reaction term is first tested by FEM and EFGM:

(34) $$\begin{array}{c} \frac{\partial^{2}C}{\partial x^{2}}+\frac{\partial^{2}C}{\partial y^{2}}=\frac{1}{\sigma }\frac{\partial C}{\partial t}, \end{array}$$

where *C* temperature, *σ* diffusion tensor, and *t* time. The analytic solution of (34) in infinite media is [34]

(35) $$\begin{array}{c} C_{t}=\frac{C_{0}}{4\pi \sigma t}\exp\left(-\frac{x^{2}}{4\sigma t}\right), \end{array}$$

where *C*_0_ is initial source in *x* = 0 at *t* = 0. The numerical simulations are initialized by the analytic solution at *t* = 1, which can be calculated from (35) with *C*_0_ = 1, and then numerical solutions are obtained in *t* = 2 in 20 × 20 area in order to approximate the effect of infinite media through a small time duration and a large enough area. Though EFGM does not always require regular nodes, it is convenient to determine the convergence rate by reducing spacing between regular nodes. The convergence behaviour of EFGM using different *d*_max⁡_ and weight functions is also evaluated. Euler forward method is applied from *t* = 1 to *t* = 2 for time integration. To find a stable RMS error in each spatial discretization, more than 10^5^ time steps of Euler method are used in our implementation. Because of regular problem domain, 20 × 20 area, the nodes of EFGM are chosen from grid points from 20 × 20 grids to 80 × 80 grids; that is, the spacing *h* is from 1 to 0.25. These grids are also used as background mesh for EFGM, respectively; for example, for 20 × 20 grids, there are 21 × 21 nodes for EFGM and 20 × 20 cells in the background mesh, and for 80 × 80 grids, there are 81 × 81 nodes for EFGM and 80 × 80 cells in the background mesh. In all the cells of background mesh, 4 × 4 Gaussian quadrature scheme is applied. The same background meshes are used as meshes of linear FEM, and the convergence curve of linear FEM is obtained using the same Gaussian quadrature scheme for fair comparison. The convergence curves of EFGM are displayed in Figure 4 along with the convergence curve of linear FEM. When *d*_max⁡_ = 1.1, both curves of cubic weight function and quartic weight function in EFGM show almost identical convergence behaviour as linear FEM. Without changing linear basis and nodal positions in EFGM, the convergence rates of EFGM become better in both weight functions when *d*_max⁡_ increases from 1.1 to 3.0, and these curves are far below the curve of linear FEM. However, the convergence behaviours of EFGM do not become better when *d*_max⁡_ has even bigger value. When *d*_max⁡_ = 4.0, the slopes of convergence curves (Figure 4) are larger, but RMS errors increase sharply in coarse nodes. A great value of *d*_max⁡_, that is, oversized influence domain, will produce oversmoothing effect as one huge element or too coarse mesh in FEM, which is the reason that RMS errors of EFGM increase largely in coarse nodes with too bigger value of *d*_max⁡_. Hence the suggested range of *d*_max⁡_ is between 1 and 3 [24, 25]. As shown by all the convergence curves in Figure 4, EFGM shows much better behaviour than linear FEM. Higher-order Gaussian quadrature scheme of each cell of background mesh will help EFGM gain better accuracy, but lower-order Gaussian quadrature scheme in the cells of finer background meshes also works quite well. Another experiment, with 2 × 2 Gaussian quadrature scheme in each cell of background mesh and total number of cells being 4 times as large as before, is compared to previous results in Figure 4. The convergence behaviours of lower-order Gaussian quadrature scheme in finer mesh (indicated by 2 × 2 in one cell in Figure 5) are better than higher-order Gaussian quadrature scheme in coarse mesh (indicated by 4 × 4 in one cell in Figure 5). 

An analytic result of reaction-diffusion system of cardiac electrical activity seldom exists that allows the performance of numerical methods to be verified. However, an analytic solution of conduction velocity in a one-dimensional fiber is available when a cubic polynomial ionic current model is used as a reaction term of the monodomain model [35]. The conduction velocity is determined by each location's activation time, which is defined by the time at which the maximum upstroke velocity occurs [3]. The cubic polynomial ionic current model is given by

(36) $$\begin{array}{c} I_{\text{ion}}=g\left[v_{m}\left(1-\frac{v_{m}}{{\text{v}}_{\text{th}}}\right)\left(1-\frac{v_{m}}{v_{p}}\right)\right], \end{array}$$

and the analytic conduction velocity *γ* is given by

**Figure 6:**
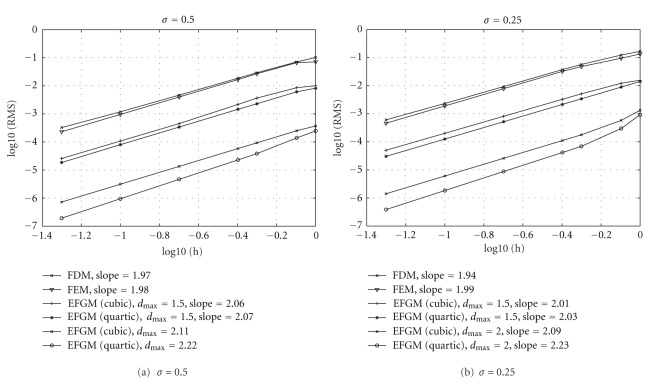
Results of conduction velocity using FDM, FEM, and EFGM. *h* is the spacing between nodes and RMS is the error measure. It shows that EFGM still has better convergence rate after the reaction term is included than FEM and FDM. 2 × 2 Gaussian quadrature scheme in each cell of one finer background mesh is applied.

(37) γ=gσAmCm2(S2S+1),S=vp2vth−1,

where *g* is the membrane conductance.  *v*_th_ and  *v*_*p*_ represent the threshold potential and the plateau potential, respectively. All the potential variables in cubic polynomial ionic current model are expressed as deviations from the resting potential. The parameters used in cubic current model are listed in Table 1. Again, the same setting of nodes is used in FDM, linear FEM, and EFGM (cubic weight function and quartic weight function), and time integration is solved by Euler forward method again. After activation times of all nodes are available, RMS error of conduction velocity can be calculated. In Figure 6, the relation between RMS errors of different numerical methods and spatial discretization is displayed. The convergence behaviours of EFGM are still better than conventional methods, FDM and FEM, after a cubic polynomial reaction term is included. In Table 2, that the computational costs to reach a similar level of error for conduction velocities of different *σ* values are shown. From Table 2, it can be seen that EFGM could achieve similar level of error using considerably less time. The computational costs presented in Table 2 have been split into “assemble” (the time taken to assemble the global system of equations) and “propagation” (the time taken to solve the global system of equations) times.

To explore the further ability of EFGM in simulation of cardiac electrical activity, one published monodomain model, a modified FHN model [7], is solved by EFGM. This FHN model [7] is

(38) $$\begin{array}{c} \frac{\partial v_{m}}{\partial t}=f\left(v_{m},I_{\text{ion}}\right)+\nabla \cdot \left(D\nabla v_{m}\right),\\ \frac{\partial I_{\text{ion}}}{\partial t}=b\left(v_{m}-dI_{\text{ion}}\right),\\ f\left(v_{m},I_{\text{ion}}\right)=c_{1}v_{m}\left(v_{m}-a\right)\left(1-v_{m}\right)-c_{2}v_{m}I_{\text{ion}}, \end{array}$$

with natural boundary condition (**D**∇*v*_*m*_) · *n* = 0. Values of parameters are taken from [7], which are listed in Table 3. State variable *v*_*m*_ is the excitation variable which corresponds to the transmembrane voltage, *I*_ion_ is the recovery current variable, *n* is the normal of the boundary, *f*(*v*_*m*_, *I*_ion_) is the excitation term, *a*, *b*, *c*1, *c*2, and *d* are parameters that define the shape of action potential. These parameters are constant over time but not necessary in space. The changes of state variables are determined by the excitation term *f*(*v*_*m*_, *I*_ion_) and diffusion term ∇·(**D**∇*v*_*m*_), and **D** is defined in (5). 

**Figure 7:**
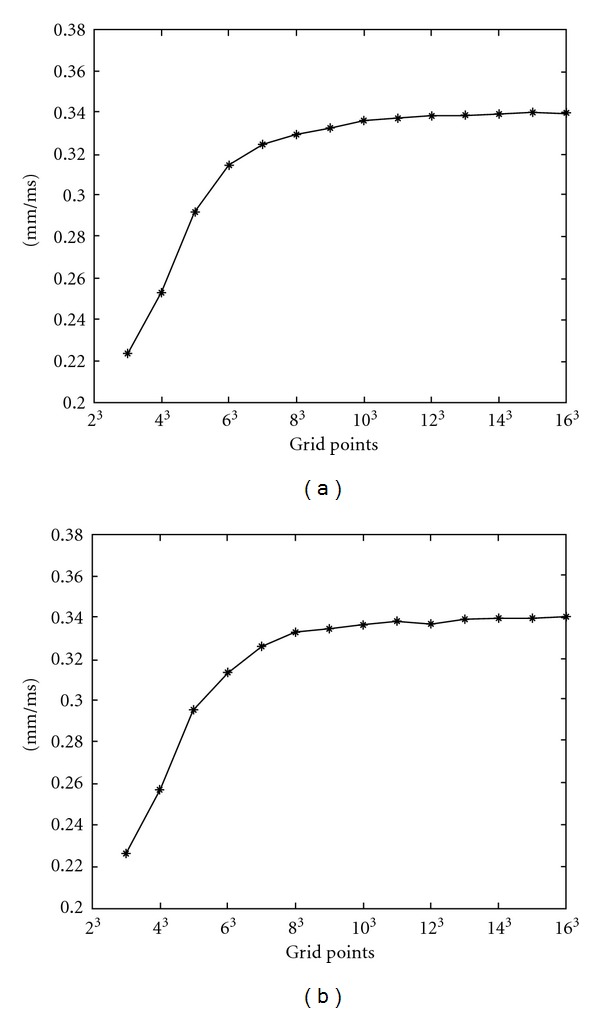
The convergence of the velocity of propagation wave with increasing density of regular sample nodes. (a) 2 × 2 × 2 quadrature points in each background cell; (b) 3 × 3 × 3 quadrature points in each background cell.

In order to find out a proper density of sample nodes in EFGM for a stable propagation wave of the FHN model in heart, two series of isotropic plane waves of electrical propagation with increasing regular sample nodes in a cube, whose size is 60 mm × 60 mm × 60 mm, are solved by setting an initial potential, 0.5, to one side of cube, and then the conduction velocity is calculated by activation time. A fourth-order Runge-Kutta method, which can select time step automatically, is applied for time integration. Two series of the isotropic electrical propagation with regular sample nodes, which change from 3 × 3 × 3 grid nodes to 16 × 16 × 16 grid nodes, and correspondingly the regular background mesh, whose background cells change from 2 × 2 × 2 to 15 × 15 × 15, are simulated, but one uses 2^3^ quadrature points in each background cell and the other uses 3^3^ quadrature points in each background cell. Convergence curves of conduction velocity are plotted in Figure 7, and a stable speed of propagation wave is achieved in both curves after sample nodes are equal to or greater than 10 × 10 × 10. 

**Figure 8:**
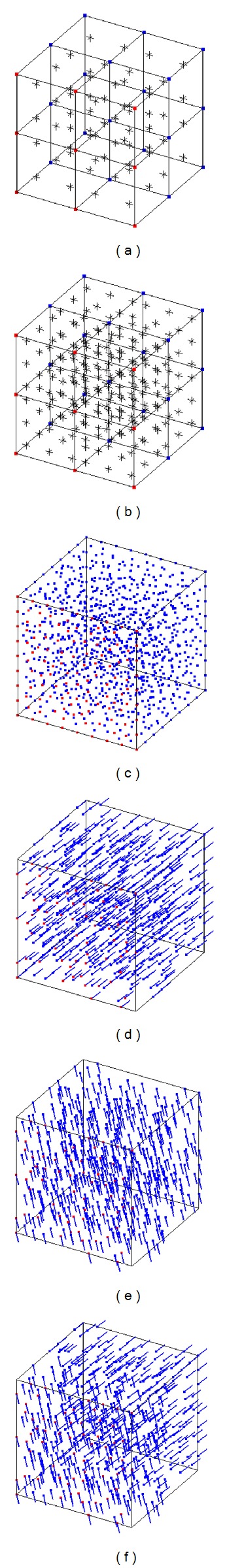
(a) 3 × 3 × 3 grid points (solid) and 2^3^ quadrature points (stars) in each background cell; (b) 3 × 3 × 3 grid points (solid) and 3^3^ quadrature points (stars) in each background cell; (c) meshfree representation of cube with irregular sample nodes (1106 nodes); (d) all the fiber directions are (0.57735, 0.57735, −0.57735); (e) all the fiber directions are (0.57735, −0.57735, 0.57735); (f) half is (0.57735, 0.57735, −0.57735) and half is (0.57735, 0.57735, −0.57735). Red points in the front side are stimulated at the beginning.

**Figure 9:**
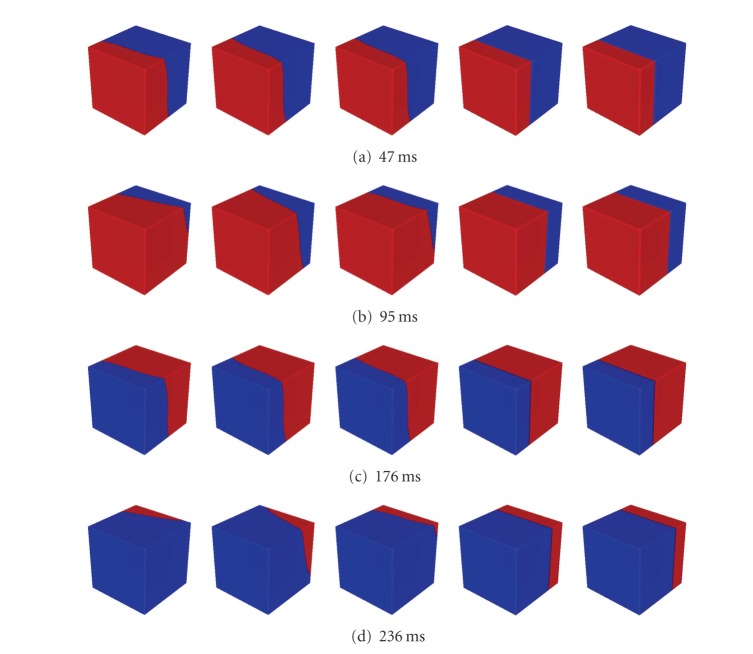
Propagation wave at 47 ms, 95 ms, 176 ms, and 236 ms with regular sample nodes (10 × 10 × 10 nodes). Fiber orientation from column 1 to column 5: (0.57735, 0.57735, −0.57735); (0.57735, −0.57735, 0.57735); half is (0.57735, 0.57735, −0.57735) and half is (0.57735, 0.57735, −0.57735); isotropic; isotropic. Except 3^3^ quadrature points in each background cell are applied in column 5, 2^3^ quadrature points in each background cell are applied in other columns. In each column total 10^3^ background cells are used for integration of Galerkin weak form. Diffuse parameter: column 1, 2, and 3 : *d*_*f*_ = 4, *d*_cf_ = 1, column 4 and 5: *d*_*f*_ = *d*_cf_ = 1. Red color represents active state and blue color represents quiescent state.

**Figure 10:**
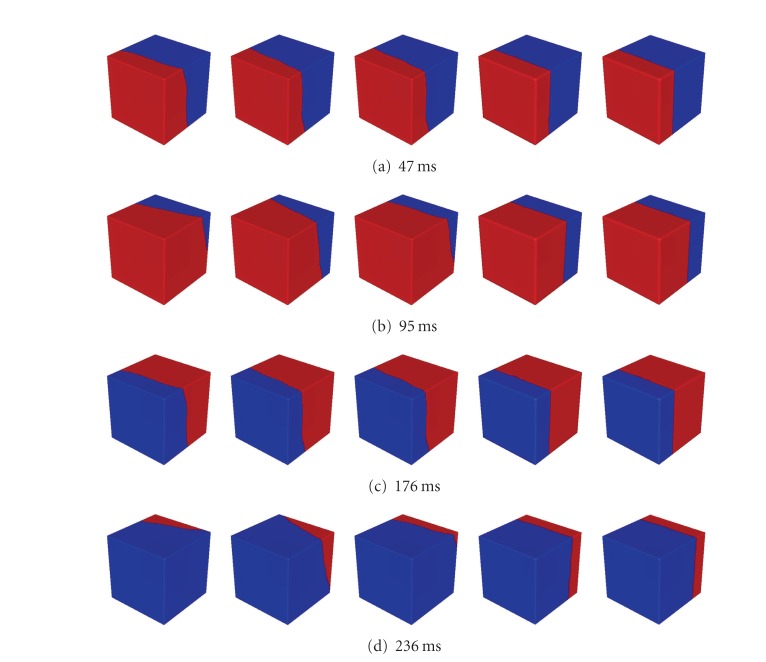
Propagation wave at 47 ms, 95 ms, 176 ms, and 236 ms with irregular sample nodes (1106 nodes). Fiber orientation from column 1 to column 5: (0.57735, 0.57735, −0.57735); (0.57735, −0.57735, 0.57735); half is (0.57735, 0.57735, −0.57735) and half is (0.57735, 0.57735, −0.57735); isotropic; isotropic. Except 3^3^ quadrature points in each background cell are applied in column 5, 2^3^ quadrature points in each background cell are applied in other columns. In each column total 10^3^ background cells are used for integration of Galerkin weak form. Diffuse parameter: column 1, 2 and 3: *d*_*f*_ = 4, *d*_cf_ =1, column 4 and 5: *d*_*f*_ = *d*_cf_ = 1. Red color represents active state and blue color represents quiescent state.

In Figure 9 propagations with different fiber orientations using 10 × 10 × 10 regular sample nodes are displayed in different time instants. A fourth-order Runge-Kutta method, which can select time step automatically, is still used for time integration. The fiber orientations from column 1 to column 3 are illustrated from Figure 8(d) to Figure 8(f). In these first three columns *d*_*f*_ is set to 1, and *d*_cf_ is set to 4. In column 4 and column 5 isotropic propagations, but different quadrature points, are displayed. In Figure 10 propagations with 1106 irregular sample nodes are displayed in different time instants. In Figure 8(c) the positions of these irregular sample nodes are shown. In Figure 10 fiber orientations in the first three columns are the same as the fiber orientations in the first three columns in Figure 9 accordingly. Two isotropic propagations with different quadrature points are also tested in irregular sample nodes, which are displayed in column 4 and column 5 of Figure 10. From Figures 9 and 10, almost identical propagations can be seen between corresponding two columns, which demonstrate that the performance of EFGM in solving FHN model will not be damaged by using irregular nodes.

**Figure 11:**
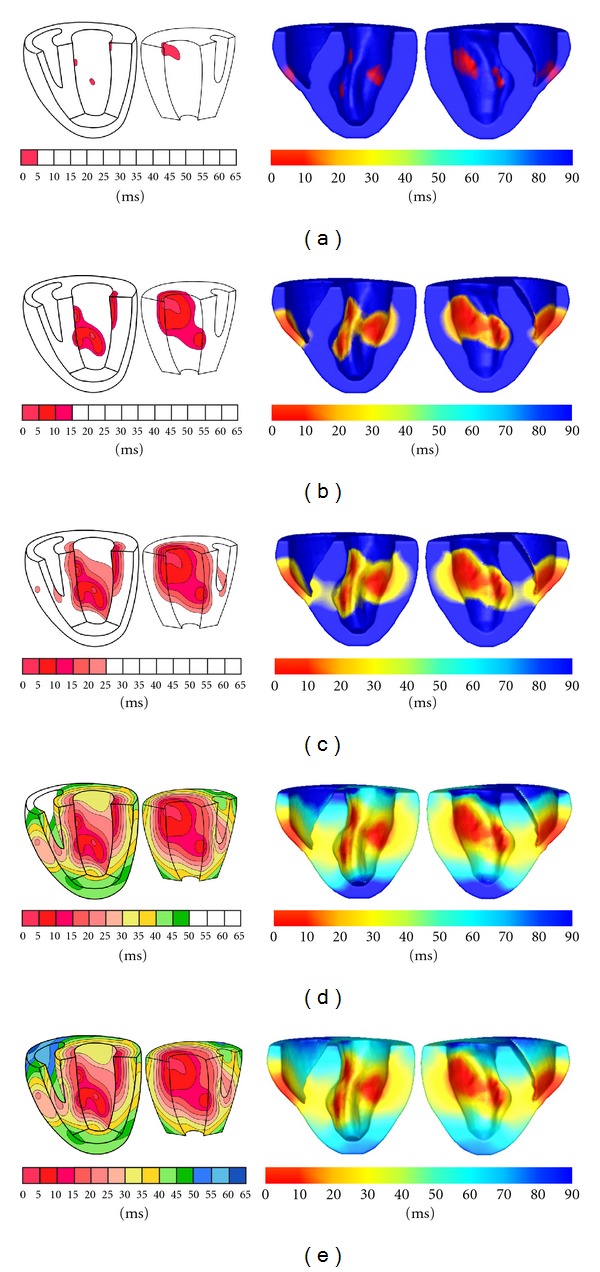
Comparison between Durrer's measurements and our simulation results. (a) 5 ms (left) and 10 ms (right), (b) 15 ms (left) and 30 ms (right), (c) 25 ms (left) and 40 ms (right),  (d) 50 ms (left) and 70 ms (right), (e) 65 ms (left) and 90 ms (right).

Finally we select 3164 sample nodes from Auckland heart model and use 3^3^ quadrature points in each background cell as suggested by the experiment mentioned previously (Figure 7). In Auckland heart model,*σ*_*f*_ is set to 4 and *σ*_*cf*_ is set to 1 as we did in the cube. Purkinje network is manually chosen on endocardium because of unavailable Purkinje network locations. From Figure 11 ((with permission): http://www.bem.fi/book/) which is generated from Durrer's [36] measurements from isolated human hearts, it can be seen that propagation of electrical activity starts from several locations on the endocardium, that is, Purkinje network extremities. Hence, a small set of nodes (around 6 nodes) around corresponding locations on the endocardium of Auckland heart model are initialized with a starting potential, 0.5 in our simulation, and the result solved by EFGM is displayed in Figure 11 in different time instants. It is reported that isolation of the heart would lead to an increase in conduction velocity [36]. Actually durations of *QRS* waveforms in healthy individuals vary from 80 ms to 100 ms since durations of *QRS* waveforms are determined by depolarization processes in the healthy hearts. That is the reason that the duration of propagation in Durrer's data is shorter than the duration of propagation in our simulation. The activation process in our simulation is qualitatively close to the published measurements as we can see in Figure 11. Once cycle of simulate of electrical propagation in Auckland heart model includes generating sample nodes, assembling of matrices and time integration. The time integration is done by the Runge-Kutta method using automatic time step. It takes 21 minutes to simulate the whole cycle of electrical propagation in Auckland hear model. 

**Figure 12:**
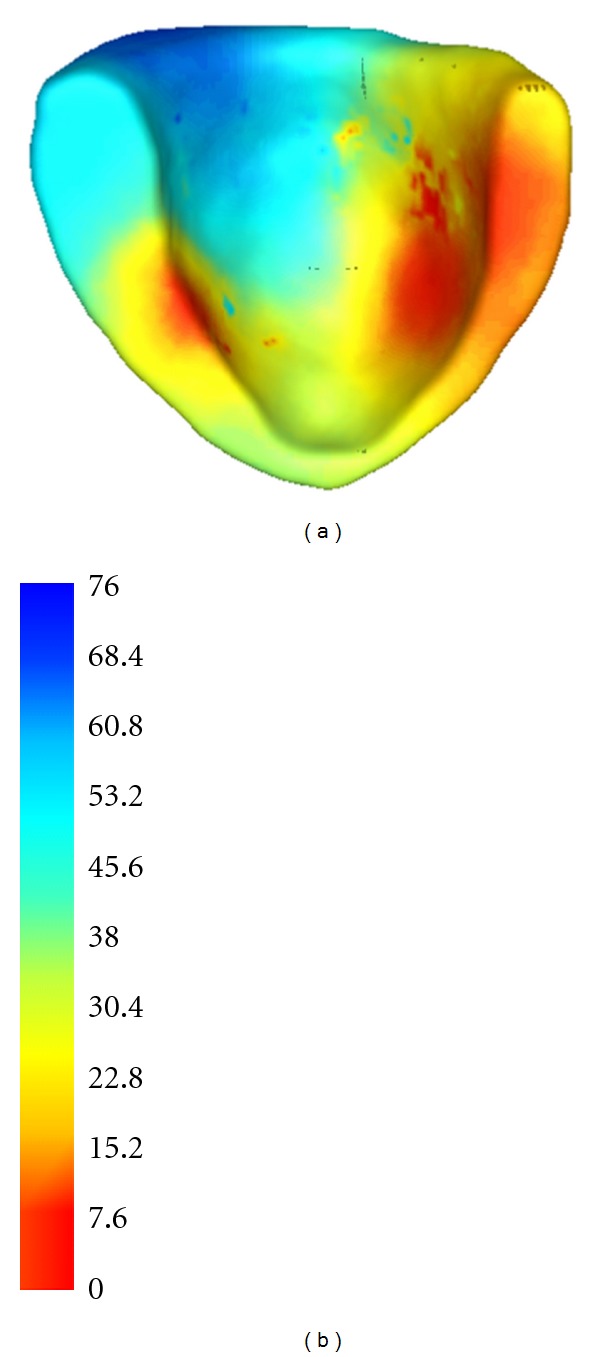
Simulation of electrical propagation in a human left ventricle (a) and its color map (b).

In the end, we also simulate the propagation in the human left ventricle extracted from digitized virtual Chinese dataset [37]. In this simulation, we only demonstrate the ability of EFMG in simulating in different cardiac geometry because of the lack of ground truth. The results are displayed in Figure 12.

## 5. Discussion

 In this paper, a numerical method without mesh constraint, EFGM, is adopted to solve reaction-diffusion equation for simulating cardiac electrical activity. This work was motivated by the successes of EFGM in mechanical modellings [24], but in our implementation, more aspects of EFGM, including the effects of influence domain (*d*_max⁡_), weight function, and integration scheme, in solving reaction-diffusion equations are evaluated.

 One of main attractions of EFGM is the meshfree particle presentation, which provides not only a convenient representation, particles without predefined connectivity, of cardiac geometry and fiber orientation, but also high interpolation accuracy for dynamical process. Our tests show that convergence behaviour of EFGM will be mainly affected by the size of influence domain. In a certain range, that is, from 1 to 3 for *d*_max⁡_, the slope of convergence curve will increase along with the value of *d*_max⁡_. However, too small size of influence domain will cause singularity in system matrices, and too large size of influence domain will also introduce large error in coarse nodes and increase the assembling cost hugely in dense nodes. We also found that EFGM performance could be minimally affected by nodal positions in simulation of propagation if the nodal density does not change largely. Different weight functions also affect the accuracy of EFGM, but the performance of cubic weight function and quadratic weight function is closed, which could be selected upon user's opinion. The numerical integration of EFGM is only evaluated on a popular regular background mesh in this paper though some works proposed irregular background meshes [24, 26], because the performance of EFGM on regular background mesh is already good enough. Especially in 3D, a lower-order Gaussian quadrature scheme in one cell of regular and fine background mesh not only saves time in assembling system matrices but also achieves rational accuracy in the irregular problem domain. Hence we would recommend regular background mesh because of easy implementation and acceptable accuracy. There has been the discussion about the construction of FEMs shape function is faster than the construction of EFGM shape function in the same spatial discretization [24], but we found that EFGM can reach a certain level of error with less computational cost than FEMs and FDMs because of higher order accuracy of EFGM shape function. Moreover, EFGM does not need to rearrange nodal positions if weight functions or polynomial bases are changed.

To fully utilize the ability of EFGM is not an easy process because wrong parameters will affect the performance of EFGM a lot, especially in 3D simulation. However, the computational cost of EFGM could be appropriately depressed by proper adjustments. First a finer background mesh with lower order quadrature, such as 2 × 2 × 2 Gauss points or even one Gauss point in one background cell, is preferable to a coarser background mesh with higher order quadrature because of cheaper computation and acceptable accuracy. Second the size of influence domain should be selected as small as possible according to local nodal density, since the time to compute shape functions and their derivatives is proportional to the number of sample nodes inside the influence domain of each Gaussian integration point. The time to assemble mass matrix and stiff matrix will also increase and the spareness of those matrix will be destroyed as result of large size of influence domain. From the point of view of accuracy, there is a minimum size of influence domain to compute the shape functions and their derivatives. In our implementation we choose a big size of influence domain first and adjust the background mesh. Then we fix the background mesh and adjust the size of influence domain. After several rounds of such adjustment, we can find suitable size of influence domain and corresponding background mesh to obtain reasonable accuracy with acceptable computational cost.

EFGM offers great potentials in simulation of cardiac behaviour, especially electrical activity because of its meshless property. This kind of numerical discretization is defined simply by placing unstructured nodes in interested area, which not only offers great convenience in implementation of adaptivity but also possibly decreases the complexity to customize the patient-specific model a lot as reasonable propagation of electrical activity in Auckland heart model could be computed in a standard desktop computer. However, further experiments with more physiological meanings, such as sustained reentry or sophisticated cellular models, in EFGM will be demanded in the future. Furthermore, a heart model with realistic geometry and components, such as with atria, ventricles, Purkinje systems, and authentic fiber structure, should be considered for better understanding of electrical activity of the whole heart.

## Figures and Tables

**Table 1:** Parameters of cubic current model.

Parameter	*v*_rest_	*v*_th_	*v*_*p*_	*g*	*C*_*m*_	*A*_*m*_
Value	−85.0 mV	−75.0 mV	15.0 mV	0.004 mS mm^−2^	0.01 *μ*F mm^−2^	200 *μ*F mm^−1^

**Table 2:** Comparison of computational costs.

	RMS	*h* (mm)	Assemble (sec)	Propagation (sec)
*σ* = 0.5				
FDM	3.30*e* − 04	0.05	n/a	258.31
FEM	2.31*e* − 04	0.05	0.20	248.26
EFGM(cubic, *d*_max⁡_ = 1.5)	4.56*e* − 04	0.2	0.11	39.50
EFGM(cubic, *d*_max⁡_ = 2.0)	2.60*e* − 04	0.8	0.05	41.33
EFGM(quartic, *d*_max⁡_ = 1.5)	3.40*e* − 04	0.2	0.07	38.83
EFGM(quartic, *d*_max⁡_ = 2.0)	1.49*e* − 04	0.8	0.04	40.60
*σ* = 0.25				
FDM	6.06*e* − 04	0.05	n/a	580.11
FEM	4.60*e* − 04	0.05	0.21	591.42
EFGM(cubic, *d*_max⁡_ = 1.5)	8.12*e* − 04	0.2	0.10	48.23
EFGM(cubic, *d*_max⁡_ = 2.0)	5.80*e* − 04	0.8	0.04	50.90
EFGM(quartic, *d*_max⁡_ = 1.5)	5.20*e* − 04	0.2	0.08	47.81
EFGM(quartic, *d*_max⁡_ = 2.0)	3.00*e* − 04	0.8	0.02	50.90

**Table 3:** Parameters of FHN model.

Parameter	*a*	*b*	*c*_1_	*c*_2_	*d*	*σ*_*f*_	*σ*_cf_
Value	0.13	0.013	0.26	0.1	1.0	4.0	1.0
